# A non-canonical ESCRT pathway, including histidine domain phosphotyrosine phosphatase (HD-PTP), is used for down-regulation of virally ubiquitinated MHC class I

**DOI:** 10.1042/BJ20150336

**Published:** 2015-09-21

**Authors:** Michael D.J. Parkinson, Siân C. Piper, Nicholas A. Bright, Jennifer L. Evans, Jessica M. Boname, Katherine Bowers, Paul J. Lehner, J. Paul Luzio

**Affiliations:** * Department of Clinical Biochemistry, Cambridge Institute for Medical Research, University of Cambridge School of Clinical Medicine, Wellcome Trust/MRC Building, Biomedical Campus, Hills Road, Cambridge, CB2 0XY, U.K.; † Department of Medicine, Cambridge Institute for Medical Research, University of Cambridge School of Clinical Medicine, Wellcome Trust/MRC Building, Cambridge Biomedical Campus, Hills Road, Cambridge, CB2 0XY, U.K.

**Keywords:** endocytosis, endosomal sorting complex required for transport (ESCRT), histidine domain phosphotyrosine phosphatase (HD-PTP), histidine-domain protein tyrosine phosphatase non-receptor type 23 (PTPN23), lysosome, ubiquitination (ubiquitylation)

## Abstract

The Kaposi's sarcoma-associated herpes virus (KSHV) K3 viral gene product effectively down-regulates cell surface MHC class I. K3 is an E3 ubiquitin ligase that promotes Lys^63^-linked polyubiquitination of MHC class I, providing the signal for clathrin-mediated endocytosis. Endocytosis is followed by sorting into the intralumenal vesicles (ILVs) of multivesicular bodies (MVBs) and eventual delivery to lysosomes. The sorting of MHC class I into MVBs requires many individual proteins of the four endosomal sorting complexes required for transport (ESCRTs). In HeLa cells expressing the KSHV K3 ubiquitin ligase, the effect of RNAi-mediated depletion of individual proteins of the ESCRT-0 and ESCRT-I complexes and three ESCRT-III proteins showed that these are required to down-regulate MHC class I. However, depletion of proteins of the ESCRT-II complex or of the ESCRT-III protein, VPS20 (vacuolar protein sorting 20)/CHMP6 (charged MVB protein 6), failed to prevent the loss of MHC class I from the cell surface. Depletion of histidine domain phosphotyrosine phosphatase (HD-PTP) resulted in an increase in the cell surface concentration of MHC class I in HeLa cells expressing the KSHV K3 ubiquitin ligase. Rescue experiments with wild–type (WT) and mutant HD-PTP supported the conclusion that HD-PTP acts as an alternative to ESCRT-II and VPS20/CHMP6 as a link between the ESCRT-I and those ESCRT-III protein(s) necessary for ILV formation. Thus, the down-regulation of cell surface MHC class I, polyubiquitinated by the KSHV K3 ubiquitin ligase, does not employ the canonical ESCRT pathway, but instead utilizes an alternative pathway in which HD-PTP replaces ESCRT-II and VPS20/CHMP6.

## Abstract (precis)

Using RNAi, a non-canonical pathway of endosomal sorting complexes required for transport was identified that is responsible for sorting virally ubiquitinated MHC class I into multivesicular bodies (MVBs) during down-regulation of this protein from the cell surface.

## INTRODUCTION

Kaposi's sarcoma-associated herpes virus (KSHV) down-regulates MHC class I molecules from the surface of infected cells through the action of virally encoded K3 and K5 ubiquitin E3 ligases, thereby allowing evasion of the host immune system [1]. KSHV K3 (KK3) promotes the polyubiquitination of MHC class I molecules through formation of a Lys^63^-linked ubiquitin chain added to a conserved lysine in the middle of the cytosolic tail of each MHC class I molecule [2–4]. Following the stable expression of KK3 in HeLa cells (HeLa-KK3 cells), newly synthesized MHC class I molecules that have trafficked through the secretory pathway to the plasma membrane are polyubiquitinated, rapidly endocytosed by a clathrin and epsin/Eps15 R-dependent route and targeted for lysosomal degradation [1,4]. This lysosomal targeting requires the endosomal sorting complex required for transport (ESCRT) proteins HRS and TSG101 [2,5].

In mammalian cells, the formation of multivesicular bodies (MVBs) commences in early endosomes following the recruitment of ESCRT proteins, which are mammalian homologues of yeast class E vacuolar protein sorting (Vps) proteins [6–8]. These proteins function both in the formation of the intralumenal vesicles (ILVs) of MVBs as well as in sorting the ubiquitinated membrane proteins into these vesicles. In yeast, structural and functional studies have led to a model in which there is sequential recruitment of ESCRTs-0, I, II and III, followed by the AAA-ATPase (ATPase associated with a variety of cellular activities) Vps4p that disassembles the ESCRT complexes from the limiting membrane of the MVB [9–11]. Yeast ESCRT-III consists of a core complex of four components (Vps2p, Vps20p, Vps24p and Vps32p/Snf7p) with four peripheral/regulatory proteins and it is the polymerization of Vps32p/Snf7p together with the co-assembly of Vps24p and Vps2p that drives ILV budding from the endosome's limiting membrane by a spring-like mechanism [12,13].

Although depletion of either the mammalian ESCRT-0 protein HRS or the ESCRT-I protein TSG101 in HeLa-KK3 cells protected MHC class I molecules from lysosomal degradation by recycling them to the cell surface [5], we observed that depletion of each of the three ESCRT-II proteins (VPS22, VPS25 and VPS36), had no effect [14]. In the present study, we examined the requirement for other ESCRT proteins in the down-regulation of KK3-polyubiquitinated MHC class I. We found that although the core ESCRT-III proteins VPS32B, VPS24 and VPS2A were required, the remaining core ESCRT-III protein VPS20/CHMP6 (charged MVB protein 6) was not needed. In contrast, the Bro1p/Vps31p-related protein HD-PTP [also known as histidine-domain protein tyrosine phosphatase non-receptor type 23 (PTPN23)] was necessary for MHC class I down-regulation. The effects of HD-PTP mutants suggested that HD-PTP acts as an alternative linker between ESCRT-I and ESCRT-III, thus indicating that a non-canonical ESCRT pathway is used to sort KK3-polyubiquitinated MHC class I into MVBs.

## EXPERIMENTAL

### Cells and antibodies

HeLa and HeLa KK3 cells were as previously described and were grown as adherent monolayers in RPMI-1640 medium (Gibco-BRL) supplemented with 10% fetal calf serum (FCS) [5].

The mouse monoclonal anti-MHC class I (w6/32) antibody was from Sigma–Aldrich, the mouse anti-MHC class I (HC10) antibody a gift from Hidde Ploegh (Whitehead Institute for Biomedical Research, Cambridge, Massachusetts, U.S.A.), the FITC-w6/32 and HRP (horseradish peroxidase)-w6/32 mouse monoclonal antibodies from Serotec, the mouse monoclonal anti-TSG101 (4A10) antibody from Gene Tex, the mouse monoclonal anti-ubiquitin (FK2) antibody from Enzo Life Sciences, the goat anti-mouse IgG (highly cross-absorbed) conjugated to Alexa Fluor 647 from Invitrogen, the mouse anti-calnexin antibody (AF8) a gift from Michael Brenner (Harvard Medical School, Boston, Massachusetts, U.S.A.), the rabbit polyclonal anti-calreticulin (PA3-900) antibody from Affinity Bioreagents, the rabbit polyclonal anti-ALIX antibody a gift from Harald Stenmark (Centre for Cancer Biomedicine, Oslo University Hospital, Montebello, N-0379 Oslo, Norway) and the rabbit polyclonal anti-GFP antibody a gift from Matthew Seaman (Cambridge Institute for Medical Research, Hills Road, Cambridge, U.K.). For immunogold EM we used rabbit polyclonal anti-Oregon green 488/FITC (A-889) antibody from Molecular Probes. Protein A conjugated to 15 nm colloidal gold was from the Department of Cell Biology, University of Utrecht, Utrecht, The Netherlands.

To generate the rabbit polyclonal antibody to human VPS20/CHMP6 (coded 8086), a GST–VPS20 fusion protein was expressed in bacteria and purified in the manner described previously [15] and used to immunize rabbits at Harlan Sera-Lab Ltd.

### siRNA knockdowns

The siRNA oligonucleotides used were siGENOME SMART or ON-TARGET plus pools from Dharmacon as follows: HRS, L-016835-00; TSG101, M-003549-01; VPS25, M-005201-00; VPS20, D-005060-00; VPS32A, M-020698-00; VPS32B, M-018075-00; VPS24, M-004696-01; VPS2A, M-020247-00; VPS2B, M-004700-00; HD-PTP, M-009417-01 (and single duplexes oligo3, D-009417-03 and oligo4, D-009417-04). For ALIX the siRNA duplex was as previously used [14]. For the VPS20 rescue experiment the siRNA was oligo1 with the sense sequence 5′-UCACCCAGAUCGAAAUGAAUU and for the HD-PTP rescue experiment the siRNA was oligo2 described by Doyotte et al. [16], with the sense sequence 5′-GCAAACAGCGGAUGAGCAAUU.

The siRNA double transfection protocol with cells harvested on day 5 was as described by Motley et al. [17] and used previously [14], except that medium containing 20% FCS was added immediately following transfection, not after 4 h. For TSG101 knockdown, cells were transfected on days 1 and 2 and harvested on day 4 to prevent extensive cell death experienced with the 5 day protocol. For CHMP2A/VPS2A knockdown, cells were only transfected on day 1 because long-term depletion of this protein caused cell death. Knockdown was assessed either by immunoblotting following SDS/PAGE as described previously [14], with molecular mass protein markers from NewEngland Biolabs, GE Healthcare Life Sciences and Bio-Rad, or by real time quantitative PCR. Ambion® Cells-to-C_T_™ kits were used for mRNA extraction and cDNA conversion, followed by TaqMan® gene expression assays for real time PCR, with TaqMan® primer/probe sets from Life Technologies including: VPS24, VPS32B, HD-PTP. Quantification of transcripts was according to Larionov et al. [18].

For the VPS20 rescue experiment, myc-VPS20 containing three silent mutations in the region of sequence identity with oligo1 siRNA was cloned into pIRESneo2 and the resulting plasmid used to transfect HeLa cells with TransIT-HeLa Monster*®* followed by antibiotic selection of stably expressing cells. Subsequent treatment of these cells with oligo1 or the ON-TARGET plus pool siRNA for VPS20 was as described above.

For the HD-PTP rescue experiments, plasmids containing oligo2 siRNA-resistant or -sensitive DNA sequences encoding wild-type (WT) and L202D/I206D mutant HD-PTP [16], were a gift from Philip Woodman (Faculty of Life Sciences, University of Manchester, Manchester, U.K.) and the HD-PTP-encoding DNA sequences were amplified and cloned into pEGFP-C3. A single knockdown transfection protocol was used. HeLa KK3 cells were transfected on day 1 with oligo2 as normal, but 12 h later were transfected with pEGFP-C3 plasmid using Effectene from Qiagen. The transiently transfected cells were harvested on day 4.

### Flow cytometric analysis

Cells were harvested, incubated in suspension with anti-MHC Class I w6/32 antibody and goat anti-mouse IgG conjugated to Alexa Fluor 647 before analysis using a FACScalibur (BD Bioscience), as previously described [14]. Control incubations were with the secondary goat anti-mouse IgG conjugated to Alexa Fluor 647 alone. To compare the effects of knockdowns in different experiments, FlowJo software was used to calculate the geometric mean of the fluorescence intensity peak for each particular knockdown and compared with a mock knockdown. Paired *t* tests were used for statistical comparison. For the HD-PTP rescue experiments, GFP-positive cells were gated as those cells with a higher green fluorescence than untransfected HeLa-KK3 cells.

### Pulse-chase labelling

Radiolabelling and immunoprecipitation of MHC class I was as previously described [5,19]. In brief, after depletion of individual ESCRT proteins with siRNA, HeLa-KK3 cells were labelled for 10 min at 37°C with (^35^S) cysteine/(^35^S)-methionine using EasyTag™EXPRESS35S Protein Labeling Mix from Perkin Elmer, followed by incubation at 37°C for 3 h in chase medium lacking radioactive amino acids. Samples were removed at 0 min, 45 min or 3 h. Following lysis with 1% Triton X-100, primary immunoprecipitation with the conformation-specific mouse monoclonal anti-MHC class I (w6/32) was followed by denaturation in 1% SDS and re-immunoprecipitation with the ‘non-conformational’ anti-MHC class I mouse monoclonal antibody HC10 and subsequent SDS/PAGE and autoradiography.

### Antibody uptake and EM

**Figure 1:**
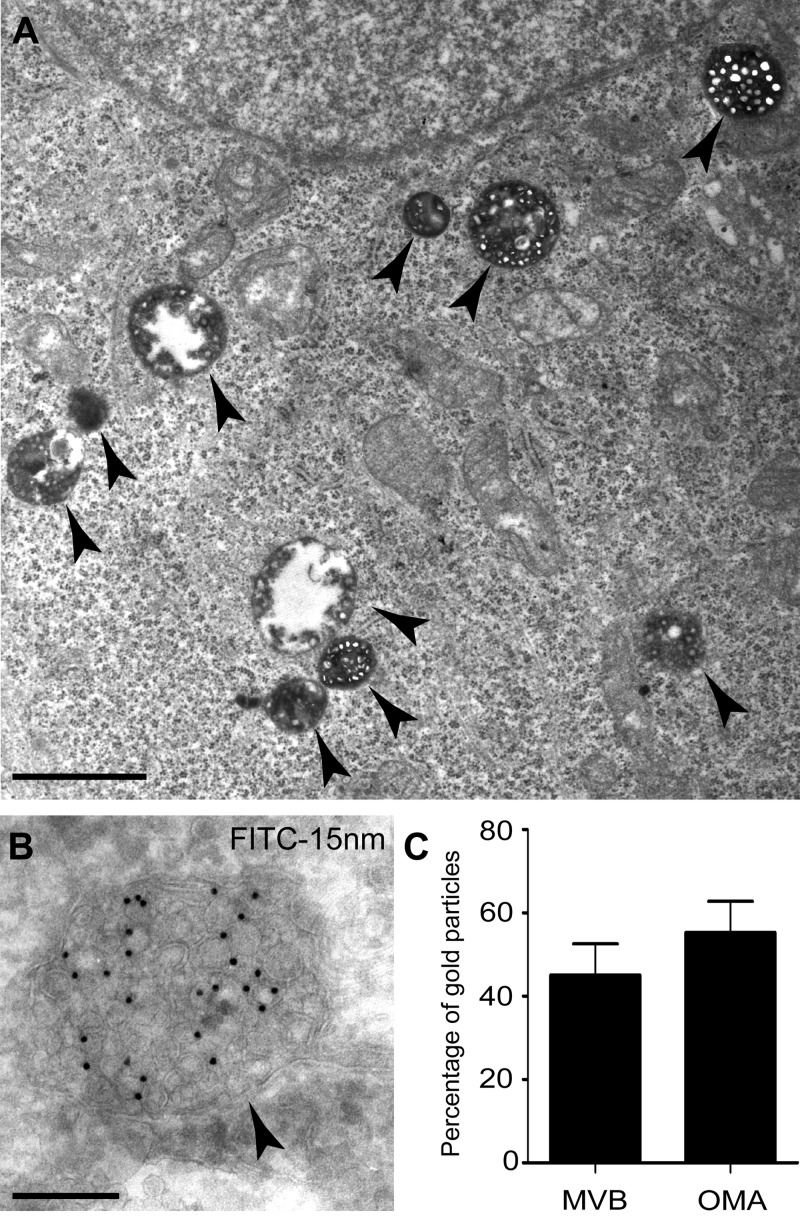
MHC class I molecules from the cell surface of HeLa-KK3 cells are endocytosed into MVBs but not the TGN (**A**) HeLa-KK3 cells treated with IFNγ and incubated with HRP-conjugated anti-MHC class I for 90 min at 37°C were fixed, incubated with DAB/H_2_O_2_ and processed for EM. Endocytosed HRP-labelled anti-MHC class I antibodies accumulated in MVBs (arrowheads), as revealed by the electron dense HRP/DAB reaction product. Scale bar, 1 μm. (**B**) HeLa-KK3 cells were treated with IFNγ, incubated with FITC-conjugated anti-MHC class I for 90 min at 37°C and frozen ultrathin sections were immunolabelled with anti-FITC (15 nm gold). A representative MVB is shown containing accumulated endocytosed anti-MHC class I antibodies (arrowhead). Scale bar, 200 nm. (**C**) Quantification of immunogold labelled FITC-conjugated anti-MHC class I on frozen ultrathin sections after uptake for 90 min at 37°C. Mean ± S.E.M. of gold particles counted. Abbreviation: OMA, other membrane-associated gold particles.

For the antibody uptake studies shown in Figure 1, HeLa-KK3 cells grown on glass coverslips in RPMI-1640 medium were pre-treated overnight at 37°C with IFNγ (200 units/ml Peprotech EC) to increase the concentration of cell surface MHC class I [20]. This pre-treatment had no effect on cell morphology. For all antibody uptake studies, cells in RPMI-1640 were incubated with either HRP-w6/32 or FITC-w6/32, initially for 2 h at 0°C followed by incubation for 90 min at 37°C. The 90 min incubation was selected to ensure loading of late endosomal compartments, following preliminary immunofluorescence microscopy experiments (result not shown). More than 90% of w6/32 bound to cell surface MHC class I at pH 7.4 remained bound when the medium was acidified to pH 5.5 and the presence of HRP or FITC did not interfere with antibody uptake when compared with the uptake of unlabelled w6/32 by immunofluorescence confocal microscopy (result not shown), consistent with labelled w6/32 being used to monitor traffic of MHC class I through endosomes. Cells incubated with HRP-w6/32 were subsequently washed with PBS at room temperature, fixed with 2% paraformaldehyde/2.5% glutaraldehyde in 0.1 M sodium cacodylate buffer for 1 h at room temperature, washed in PBS, incubated with DAB (3,3′-diaminobenzidine)/H_2_O_2_ (1 mg/ml DAB; 4 μl of H_2_O_2_ in 10 ml of PBS) for 10 min in the dark at room temperature and processed for transmission EM as described previously [21]. For immunogold EM, cells incubated with FITC-w6/32 were washed with PBS, fixed with 4% paraformaldehyde/0.1% glutaraldehyde in 0.1 M sodium cacodylate buffer for 1 h at room temperature, processed and frozen ultrathin sections (50–70 nm) prepared and labelled with anti-FITC and gold-conjugated protein A as previously described [21]. For quantification, MVBs were defined as membrane-bound vacuoles that contained more than one internal vesicle. Following uptake of FITC-w6/32 into HeLa-KK3 cells, 7839 gold particles were counted in total on six independently labelled EM grids from two separate experiments and after VPS20 depletion 1535 gold particles were counted on three independently labelled EM grids.

## RESULTS

### Endocytosed polyubiquitinated MHC class I is delivered to MVBs

The previously established requirement for HRS and TSG101 in down-regulating cell surface MHC class I and targeting polyubiquitinated MHC class I for lysosomal degradation in HeLa-KK3 cells following endocytosis, implied that this protein was trafficking to lysosomes via MVBs [2,5]. To confirm this, we carried out anti-MHC class I antibody uptake experiments on HeLa-KK3 cells followed by EM analysis. We observed labelling of MVBs with electron dense reaction product after uptake of HRP-conjugated anti-MHC class I antibodies followed by incubation with DAB/H_2_O_2_ (Figure 1A). We also examined HeLa-KK3 cells by immuno–EM after endocytosis of FITC-conjugated anti-MHC class I and detection with anti-FITC antibody. Internalized anti-MHC class I was consistently observed within multi-vesicular structures, particularly on ILVs within MVBs (Figure 1B). Quantification showed that 44.9±7.7% of the anti-MHC class I was in compartments defined as MVBs after 90 min uptake of cell-surface bound anti-MHC class I antibodies (Figure 1C). The remainder was associated with other membranes, including tubular and vesicular elements, consistent with it being in the endocytic pathway.

### The effect of depletion of different ESCRT proteins on down-regulation of polyubiquitinated MHC class I

**Figure 2:**
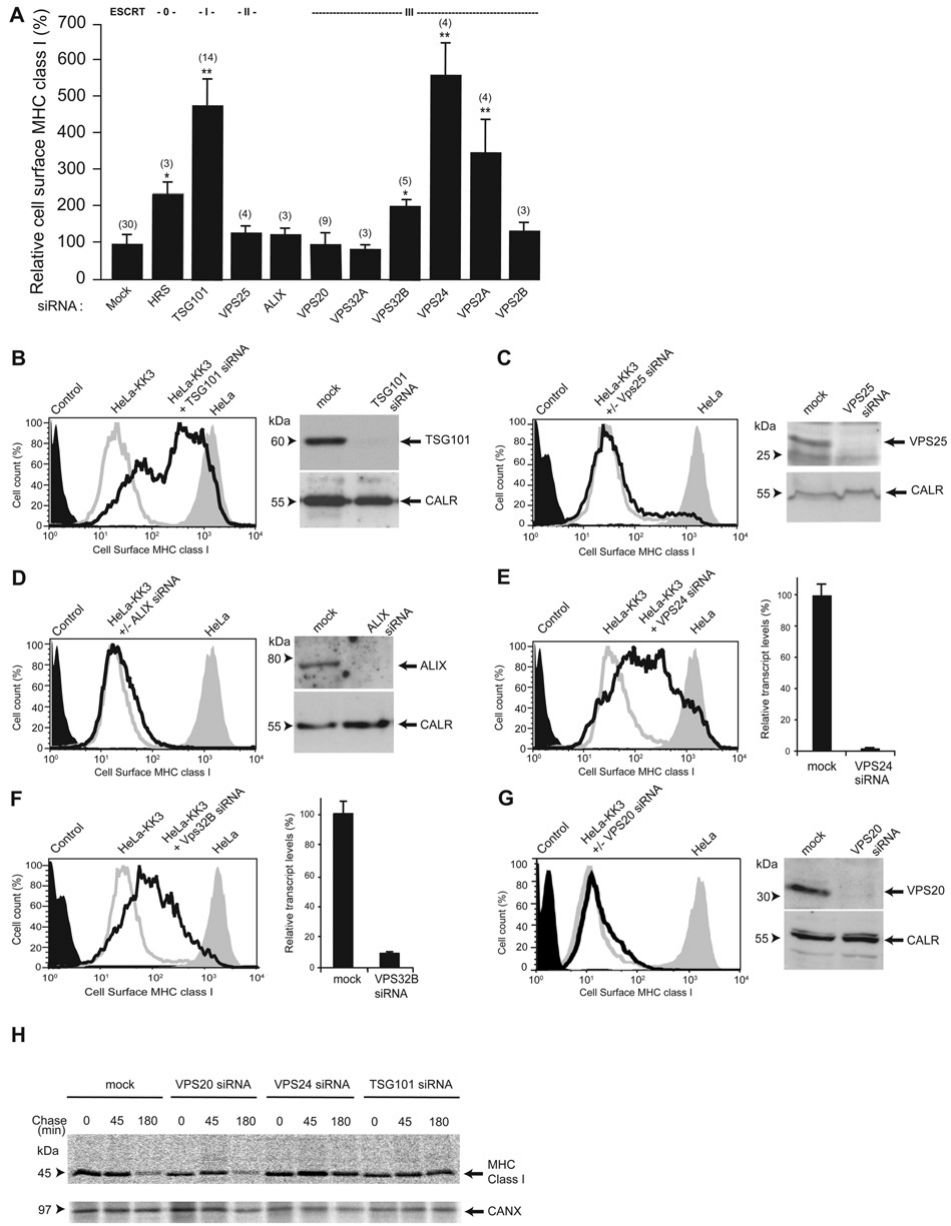
The effect of depleting ESCRT proteins on down-regulation of virally-ubiquitinated MHC class I (**A**) Summary of cytofluorometric analysis of cell surface expression of MHC class I in HeLa-KK3 cells after depletion of individual ESCRT proteins with siRNA pools. In each experiment geometric mean fluorescence data from siRNA-treated cells were compared with data from mock-treated cells, normalized to 100%. Mean ± S.E.M., number of experiments in brackets, *P*-values **≤0.01, *<0.05. (**B–G**) Representative cytofluorometry traces of cell surface expression of MHC class I in HeLa-KK3 cells following depletion of individual ESCRT proteins (unfilled black traces). Traces for secondary antibody controls (filled black traces), mock-treated HeLa-KK3 (unfilled grey traces) and HeLa (filled grey traces) cells are also shown. The effectiveness of knockdown is shown by either immunoblotting or real time quantitative PCR (mean ± S.E.M. of three samples). CALR, calreticulin. (**H**) Pulse-chase analysis of degradation of MHC class I HeLa-KK3 cells following depletion of individual ESCRT proteins. The immunoprecipitation of CANX (calnexin), with mouse anti-CANX antibody AF8 from the pulse-chase radiolabelled cell lysates provided the input lysate control.

To investigate the requirement for different ESCRT proteins in the down-regulation of KK3-polyubiqitinated MHC class I we determined the amount of MHC class I on the surface of HeLa-KK3 cells by cytofluorometric analysis after incubation with various siRNAs. In agreement with previous experiments [2,5], depletion of either the ESCRT-0 protein HRS or the ESCRT-I protein TSG101 increased the surface concentration of MHC class I (Figure 2A). This was observed as a shift in cytofluorimetry traces to the right (Figure 2B for representative individual trace after TSG101 depletion), indicating an inhibition of KK3-mediated MHC class I down-regulation. Depletion of the ESCRT-II protein VPS25 failed to increase the cell surface concentration of MHC class I (Figures 2A and 2C), as previously reported (Figure 8 in [14]) and there was also no effect of depleting ALIX (Figures 2A and 2D), a mammalian homologue of yeast Bro1p/Vps31p that can provide an alternative to ESCRT-II in linking TSG101 of ESCRT-I to VPS32/CHMP4 in ESCRT-III [22,23].

The human homologues of the core yeast ESCRT-III proteins are VPS20/CHMP6, VPS24/CHMP3, VPS2/CHMP2 and VPS32/CHMP4, but whereas the former pair each have a single mammalian isoform, VPS2/CHMP2 has two isoforms and VPS32/CHMP4 has three. Depletion of VPS24/CHMP3 (Figures 2A and 2E) and VPS2A/CHMP2A (Figure 2A), but not CHMP2B/VPS2B (Figure 2A), resulted in an increase in MHC class I on the surface of HeLa-KK3 cells. In agreement with a previous study [24], depletion of VPS32B/CHMP4B but not VPS32A/CHMP4A or VPS32C/CHMP4C specifically increased cell surface MHC class I (Figures 2A and 2F and result not shown). All the observed increases in cell surface MHC class I described above after siRNA treatment, occurred in HeLa-KK3 but not HeLa cells and were confirmed with at least two of the individual oligonucleotides making up each siRNA pool (result not shown).

Although depletion of VPS24/CHMP3 and one isoform of each of VPS2/CHMP2 or VPS32/CHMP4 in HeLa-KK3 cells caused an increase in cell surface MHC class I, no effect was seen when the fourth core subunit of ESCRT-III, VPS20/CHMP6, was depleted (Figures 2A and 2G). Moreover, pulse-chase radiolabelling experiments in HeLa-KK3 cells showed that, consistent with this observation, there was degradation of the MHC class I after depletion of VPS20/CHMP6 in contrast with the protection from degradation observed after depletion of VPS24/CHMP3 or TSG101 (Figure 2H). A similar lack of effect of depleting VPS20/CHMP6 on the stimulation of MHC class I degradation by KK3 was previously reported by Langelier et al. [25], without showing data.

### Depletion of VPS20/CHMP6 alters the morphology of endocytic compartments

**Figure 3:**
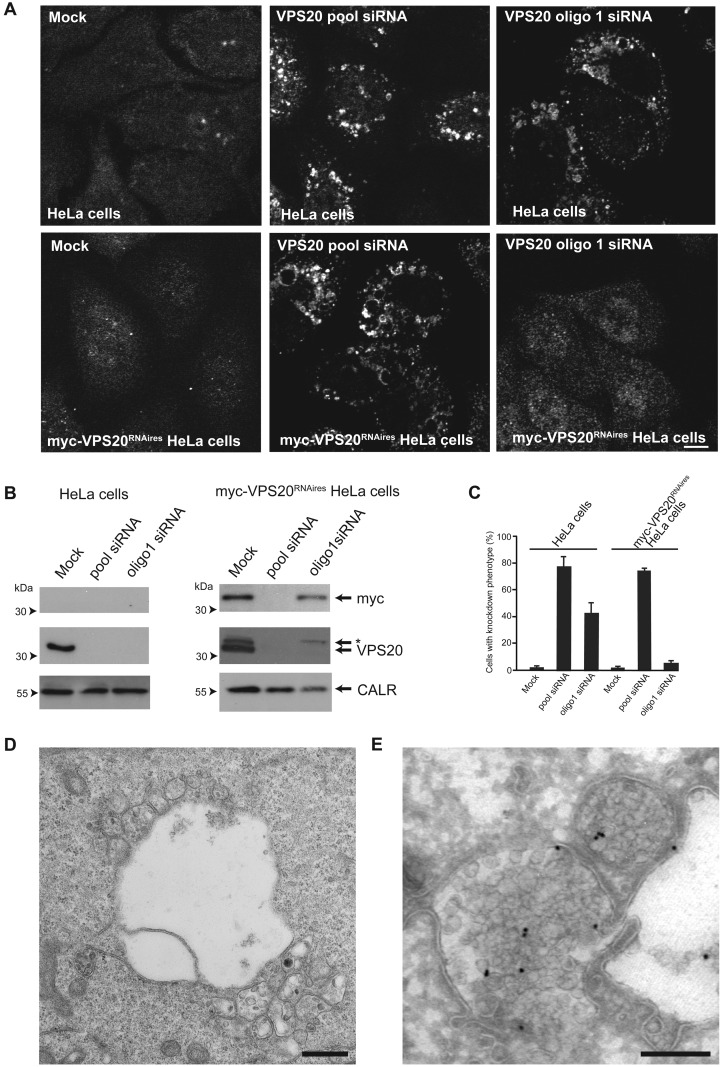
The effect of depleting VPS20/CHMP6 on endocytic compartments (**A**) Immunofluorescence confocal microscopy of HeLa cells stained with anti-ubiquitin antibodies, following mock knockdown or knockdown with either a pool of siRNAs or a single siRNA, oligo1 (upper panels). Lower panels show stably-transfected HeLa cells expressing oligo1 siRNA-resistant myc-tagged VPS20 (myc-VPS20^RNAires^ HeLa cells), after the same knockdown treatments. Scale bar=10 μm (**B**) Immunoblotting to show expression of VPS20 and myc-tagged VPS20 in the HeLa cells and myc-VPS20^RNAires^ HeLa cells following siRNA knockdown. *myc-VPS20; CALR, calreticulin. (**C**) Proportion of cells with enlarged, ubiquitinated compartment phenotype in HeLa cells or myc-VPS20^RNAires^ HeLa cells after depletion of endogenous VPS20 with either a pool of siRNAs or oligo1. Mean ± S.E.M. (three coverslips, 50 cells per coverslip). (**D**) Transmission electron micrograph of a HeLa cell treated with the VPS20 siRNA pool, showing a swollen endocytic compartment and associated MVBs. Scale bar=500nm. (**E**) Transmission electron micrograph of an anti-FITC antibody (15 nm gold)-stained frozen section from a HeLa-KK3 cell allowed to endocytose FITC-conjugated anti-MHC class I for 90 min at 37°C after knockdown of VPS20. Two representative MVBs are shown containing accumulated endocytosed anti-MHC class I antibodies. Scale bar=200nm.

In our experiments, the absence of an effect of depleting VPS20/CHMP6 on cell surface MHC class I was observed despite a profound effect on the morphology of the endosomal system with the appearance of enlarged, ubiquitinated compartments in up to 80% of the cells (Figure 3A). These enlarged, ubiquitinated compartments were also LAMP1-positive (result not shown) and were seen when either HeLa or HeLa-KK3 cells were incubated with a pool of four VPS20/CHMP6 siRNA oligonucleotides. Similar effects were seen when the cells were incubated with three single oligonucleotides from the siRNA pool (see Figure 3A for oligo1), although in individual experiments with the single oligonucleotides, the proportion of cells with enlarged, ubiquitinated compartments varied from ∼10%–70%, broadly correlating with the extent of VPS20/CHMP6 depletion assessed by SDS/PAGE of ∼40%–90% (result not shown). The enlarged, ubiquitinated compartments had some similarities to those observed previously when ESCRT proteins such as HRS or TSG101 were depleted [26,27]. Given the variability in the proportion of cells clearly showing the enlarged, ubiquitinated compartment phenotype after VPS20/CHMP6 depletion with single siRNA oligonucleotides, we designed a rescue experiment. The enlarged, ubiquitinated compartments formed after incubation with oligo1, but not the siRNA pool, could be rescued by expressing oligo1 siRNA-resistant myc-tagged VPS20/CHMP6 (Figures 3A–3C). Transmission EM showed that clusters of MVBs were often associated with the enlarged compartments (Figure 3D). Endocytosed FITC-labelled antibodies to MHC class I were still delivered to these MVBs in the VPS20/CHMP6-depleted HeLa-KK3 cells (Figure 3E), with 40.3±1.3% of the antibodies being associated with MVBs in these cells, after 90 min of antibody uptake.

### HD-PTP is required for the down-regulation of polyubiquitinated MHC class I

**Figure 4:**
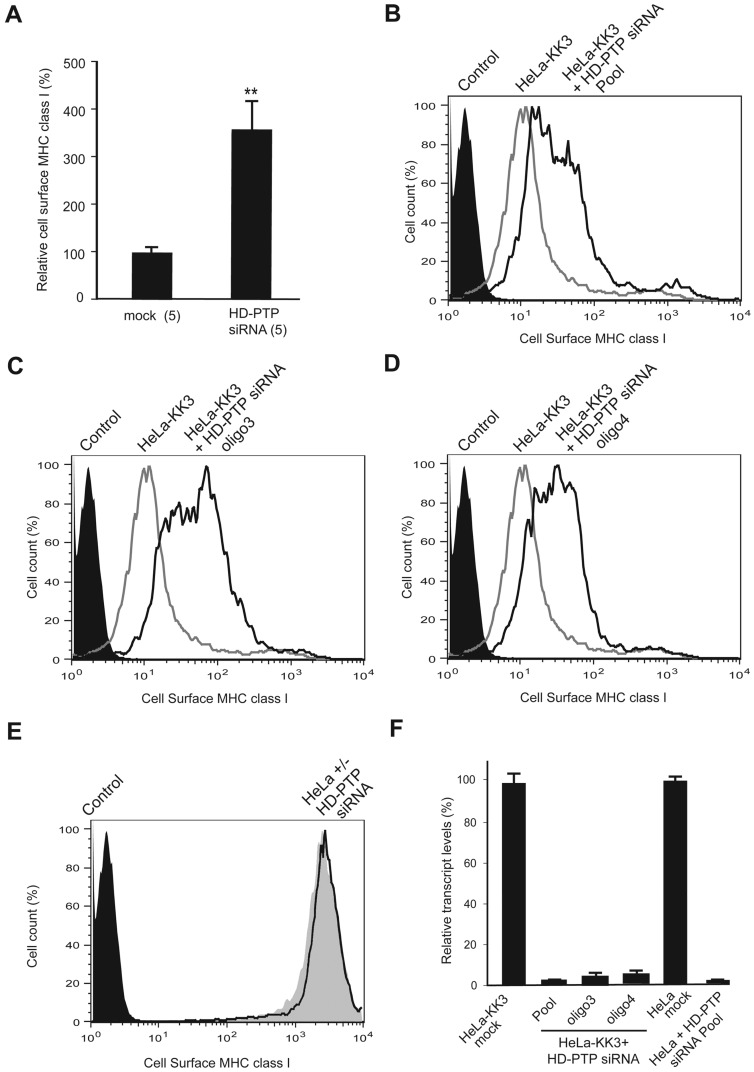
The effect of depleting HD-PTP on down-regulation of virally ubiquitinated MHC class I (**A**) Summary of cytofluorometric analysis of cell surface expression of MHC class I in HeLa-KK3 cells following mock knockdown or depletion of HD-PTP with an siRNA pool. Geometric mean fluorescence data from siRNA-treated cells were compared with data from mock-treated cells, normalized to 100%. Mean ± S.E.M., number of experiments in brackets, *P*-value **≤0.01. (**B–D**) Representative cytofluorometry traces of cell surface expression of MHC class I in HeLa-KK3 cells following depletion of HD-PTP (unfilled black traces) with an siRNA pool (**B**) or individual siRNAs from the pool, oligo3 (**C**) and oligo4 (**D**). Traces for mock treated HeLa-KK3 cells (unfilled grey traces) and secondary antibody controls (filled black traces) are also shown. (**E**) Representative cytofluorometry trace of cell surface expression of MHC class I in HeLa cells following depletion of HD-PTP with a siRNA pool (unfilled black trace). Traces for mock-treated HeLa cells (filled grey trace) and secondary antibody control (filled black trace) are also shown. (**F**) Real time quantitative PCR to show the effectiveness of knockdown in the representative experiments shown in panels (**B**–**E**). Mean ± S.E.M. of three samples.

**Figure 5:**
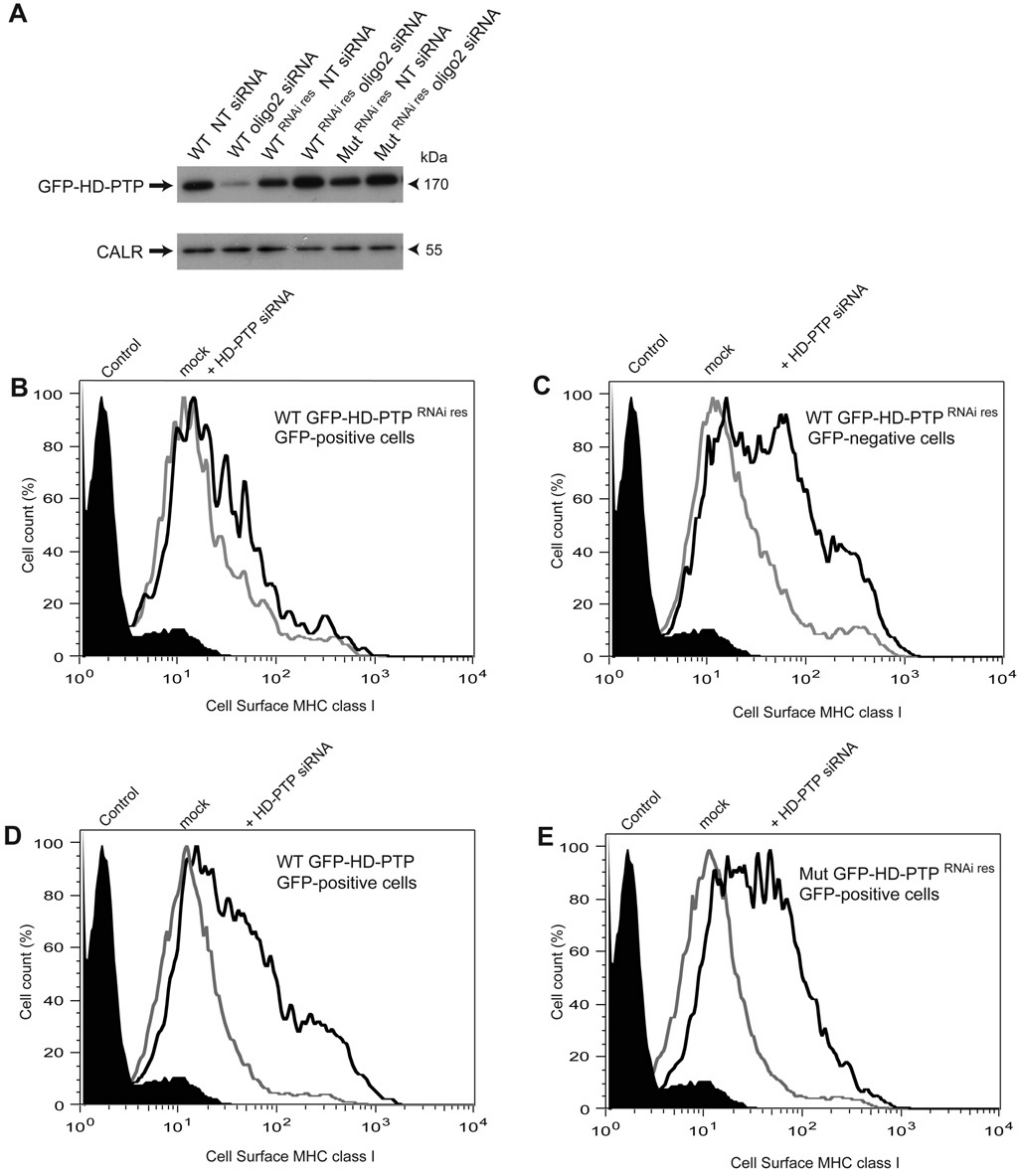
Rescuing the down-regulation of virally ubiquitinated MHC class I following knockdown of HD-PTP (**A**) HeLa-KK3 cells were treated with either non-targeting siRNA (NT) or HD-PTP oligo2 siRNA on day 1 and then 12 h later transfected with pEGFP-C3 plasmids containing inserts encoding WT, oligo2 siRNA resistant WT (WT^RNAires^) or oligo2 siRNA resistant L202D/I206D mutant (Mut^RNAires^) GFP-HD-PTP. On day 4, the presence of GFP-HD-PTP in harvested cells was detected by immunoblotting with anti-GFP. (**B–D**) Representative cytofluorometry traces of cell surface expression of MHC class I in HeLa-KK3 cells treated with oligo2 siRNA and transfected with GFP-HD-PTP constructs (unfilled black traces). In (**B** and **C**), cells were treated with oligo2 siRNA and transfected with WT GFP-HD-PTP^RNAires^ before analysing the GFP-positive (**B**) and negative (**C**) cells. In (**D**), cells were treated with oligo2 siRNA and transfected with WT GFP-HD-PTP and, in (**E**), treated with oligo2 siRNA and transfected with L202D/I206D mutated Mut GFP-HD-PTP^RNAires^. Traces for secondary antibody controls (filled black traces) and mock-treated HeLa-KK3 (unfilled grey traces) are also shown.

In the canonical ESCRT pathway found in yeast, ESCRT-II binds to Vps20p and triggers the homo-oligomerization of Vps32p/Snf7p [12]. Given that ESCRT-II, VPS20/CHMP6 and ALIX were not required for down-regulation of KK3-polyubiquitinated MHC class I in HeLa-KK3 cells we searched for another protein that could link mammalian ESCRT-I to VPS32/CHMP4 in ESCRT-III. We investigated the effect of depletion of the protein HD-PTP that is structurally related to ALIX and is a mammalian homologue of yeast Bro1p/Vps31p [28]. HD-PTP can bind to the ESCRT-I components TSG101 and UBAP1 as well as having a central Bro1 domain that can interact with CHMP4B [29,30]. In contrast with the lack of effect when depleting ALIX in HeLa-KK3 cells, the knockdown of HD-PTP with a pool of four siRNAs resulted in a significant increase in the cell surface concentration of MHC class I (Figures 4A and 4B). The effect was closest to that seen when depleting VPS2A (Figure 2A) when geometric mean fluorescence was measured over five experiments (Figure 4A). All four single oligonucleotides in the pool had similar effects (Figures 4C and 4D for oligo3 and oligo4; other results not shown) and there was no change in cell surface MHC class I when control HeLa cells were treated with the pool (Figure 4E). The pool of siRNAs and the single oligonucleotides in the pool all reduced HD-PTP transcript levels >90% (Figure 4F). We then designed a rescue experiment based on a previous study in which depletion of HD-PTP was shown to reduce the transfer of fluid phase markers and the EGFR (epidermal growth factor receptor) to lysosomes [16]. As expected, siRNA-sensitive GFP-tagged HD-PTP expressed in transiently transfected HeLa-KK3 cells was depleted by treatment with a single siRNA duplex (oligo2) but oligo2 siRNA-resistant GFP-tagged HD-PTP was not (Figure 5A). In a population of HeLa-KK3 cells transiently transfected with WT siRNA-resistant GFP-tagged HD-PTP (WT GFP-HD-PTP^RNAires^), cell surface MHC class I was analysed separately in the GFP-positive and GFP-negative cells. The cytofluorimetry trace of MHC class I in the GFP-positive cells expressing WT GFP-HD-PTP^RNAires^ showed almost no shift to the right after oligo2 knockdown, demonstrating a rescue (Figure 5B). This was in contrast with the expected rightward shift of the MHC class I cytofluorimetry trace in the GFP-negative, HD-PTP-depleted cells from the same oligo2-treated, transiently transfected population (Figure 5C). Rightward shifts of MHC class I cytofluorimetry traces, showing no rescue, were also observed in oligo2-treated, transiently transfected HeLa-KK3 cells expressing WT siRNA-sensitive GFP-tagged HD-PTP (WT GFP-HD-PTP; Figure 5D) or a siRNA-resistant GFP-tagged L202D/I206D mutant HD-PTP (Mut GFP-HD-PTP^RNAires^; Figure 5E). The L202D and I206D mutations are in the Bro1 domain and prevent binding to VPS32B/CHMP4B [16]. Thus, the data shown in Figure 5 are consistent with the binding of HD-PTP to VPS32B/CHMP4B being required for down-regulation of MHC class I in HeLa-KK3 cells.

## DISCUSSION

In the present study, we have shown that in HeLa-KK3 cells down-regulation of KK3-polyubiquitinated MHC class I involves traffic from the plasma membrane to lysosomes via MVBs. Our siRNA knockdown experiments, together with data previously published [5,14,24,25], are consistent with the down-regulation requiring ESCRT-0 (HRS), ESCRT-1 (TSG101), plus three of the four core components of ESCRT-III (VPS32B/CHMP4B, VPS24/CHMP3 and VPS2A/CHMP2A), but not ESCRT-II (VPS25) or the ESCRT-III protein VPS20/CHMP6. These data imply that the canonical ESCRT pathway in which ESCRT-II and VPS20/CHMP6 link ESCRT-I to the polymerization of the ESCRT-III protein VPS32/CHMP4 cannot account for the sorting into MVBs and down-regulation of KK3-polyubiquitinated MHC class I. This contrasts with studies showing a requirement for ESCRT-II in the down-regulation of a variety of transporters, chemokine and growth factor receptors [31,32]. However, it should be noted that ESCRT-II and VPS20/CHMP6 are not required for the ESCRT-dependent processes cytokinesis and retroviral budding from the cell surface [7,12]. In addition, Vps20p is not required for ESCRT-dependent surveillance of nuclear pore complex assembly in yeast [33] and VPS20/CHMP6 is not required for ESCRT-dependent plasma membrane wound repair [34].

Although the Bro1p-related protein ALIX was found to link ESCRT-I to ESCRT-III for the budding of the human immunodeficiency virus [25] it is not required for down-regulation of KK3-ubiquitinated MHC class I. We found that a different Bro1p-related protein, HD-PTP, is required for the down-regulation of KK3-polyubiquitinated MHC class I, consistent with a non-canonical ESCRT pathway being used for the down-regulation of this specific cargo. Interestingly, the L202D/I206D mutant of HD-PTP that cannot bind VPS32B/CHMP4B [16], was unable to rescue the effects of endogenous HD-PTP deletion on KK3-mediated down-regulation of MHC class I, despite it previously having been shown to be as effective as WT HD-PTP in supporting EGF sorting into MVBs [16]. The role of HD-PTP in sorting the EGFR appears to be more complex than simply acting as a link between ESCRT-I and ESCRT-III, since it also binds to STAM2 in mammalian ESCRT-0 and has been proposed to combine with the deubiquitinating enzyme UBPY to transfer EGFR from ESCRT-0 to ESCRT-III and help drive the sorting of EGFR into MVBs [35]. Data from experiments on *Drosophila* and/or mammalian cells has also implicated HD-PTP in the endosomal sorting of integrins, E-cadherin and Toll receptors [36–39], although in these cases its mechanism of action is less clear.

It is well recognized that the molecular machinery to form and sort ubiquitinated cargoes into MVBs in mammalian cells has more complexity and diversity than the canonical ESCRT pathway used in yeast [40]. A non-canonical ESCRT pathway independent of HRS and TSG101, but requiring ALIX and ESCRT-III proteins, has been shown to be responsible for sorting a non-ubiquitinated G protein-coupled receptor, protease-activated receptor 1, into MVBs for subsequent degradation by lysosomal hydrolases [41]. Also, there is evidence for more than one type of MVB in the same cell [42] and for non-ESCRT-mediated formation and sorting into MVBs [43–46]. In addition, studies of three mammalian ESCRT-III proteins, VPS24/CHMP3, VPS60/CHMP5 and VPS2/CHMP2B, have suggested a regulatory role in endosome–lysosome fusion, distinct from MVB formation [47–49]. The effects of depletion of VPS20/CHMP6 seen in the present study, with the accumulation of tethered MVBs associated with swollen LAMP1 positive endocytic compartments, are also consistent with a regulatory role in endosome–lysosome fusion, although they could equally imply that all ubiquitinated cargoes must be correctly sorted into MVBs if they are to become competent for fusion.

In summary, our data provide evidence of a non-canonical ESCRT pathway to sort one particular cargo, KK3-polyubiquitinated MHC class I, into MVBs in mammalian cells. This pathway utilizes HD-PTP rather than ESCRT-II and VPS20/CHMP6 in linking ESCRT-I to ESRCT-III. This difference from the ESCRT pathway used to down-regulate other cell surface proteins may reflect the homogeneity of the Lys^63^ polyubiquitin chains added to the MHC class I cytosolic tail by KK3, which contrasts with the mixture of multiple monoubiquitination and polyubiquitination and/or mixed linkage polyubiquitin chains that has been reported for the cytosolic tails of some other down-regulated membrane proteins and/or by ubiquitination with other E3 ligases [50–52].

## Abbreviations

CHMP: charged MVB protein
DAB: 3,3′-diaminobenzidine
EGFR: epidermal growth factor receptor
ESCRT: endosomal sorting complex required for transport
FCS: fetal calf serum
HD-PTP: histidine domain phosphotyrosine phosphatase
HRP: horseradish peroxidase
ILV: intralumenal vesicle
KK3: Kaposi's sarcoma-associated herpes virus protein K3
KSHV: Kaposi's sarcoma-associated herpes virus
MVB: multivesicular body
PTPN23: histidine-domain protein tyrosine phosphatase non-receptor type 23
Vps/VPS: vacuolar protein sorting
WT: wild-type
